# 

**Published:** 1887-12

**Authors:** 


					﻿BOOKS AND PAMPHLETS RECEIVED.
Facts and Fallacies in Climatology. By H. E.
Beebe, M. D.
Annual Report of the Homoeopathic Hospital of
Melbourne, Australia, for 1887.
Homoeopathic League Tract No. 15. Remarks on tne
Explanation given by Dr. Lauder Brunton.
Calendar of the Medical Faculty, University of
Toronto.
INDEX
TO THE
Homeopathic Physician.
VOLUME VII.
PAGE
Abies canadensis,  ..............444
Abies nigra,....................444
Abridged Therapeutics, Founded
upon Histology and Cellular Path-
ology. By Dr. Med. Schussler. Trans-
lated by E. H. Holbrook, M. D., Re-
view of, ........................140
Abrotanum,.......................444
Aconite..........17,49,50,53,54, 61,
115, 173, 184, 185, 201, 204, 256,261,
281, 282, 329,830, 361,369,377, 387, 433,
445,446, 447,449, 450, 451, 453, 456, 461,462
Actea rac.,.......... 52,53,101,155, 379
Aesculus, hip,............. 63,210, 406
Aethusa..............60, 61, 79,114, 211
Agaricus,........50, 51, 238, 332,455, 462
Agnus...................52, 53, 282, 446
Ailanth......... 49, 50, 52, 406, 450
Ailanthus. A. McNeil, M.D...... 456
Alcohol, ............... 354
Aletris,........................453
Allen, John V., M. D. Capsicum, A
Few Comparisons of,........	10
Rep. to Haemorrhage of Bowels, . 485
Typhoid Fever, ......... 487
Allen, Dr. T. F., Viewsupon Homoeo-
pathic Practice,	396
Allium cepa, . . . 51, 52, 61, 211,241,
375,446,450
Allium sat.,.....................336
Aloes, ......67,239,240,364,445,456
Alumen, . ............... 49
Alumina, . . . .2,47,49,50,52,53,60,
61,170,171, 199,204, 239, 407,432, 444
Alumni Association of Hahnemann
Medical College, Notice of,...... 221
Ambra, ............. 52, 53, 462
American Institute of Homoeopathy.
Ad. Lippe, M, D.,..............117
American Institute and Its Laughter.
P. P. Wells, M. D.,............107
of Homoeopathy, Transactions of, 105
American Medicinal Plants. By C. F.
Millspaugh, M. D., Review of,. . 69, 436
Ammoniacum, ............ 224
Ammon. Br., .......... 49,52, 53
Ammonium Carbonicum,. . 48,49, 50,
51, 52, 53, 60, 61,194, 281, 369, 407,425,
458, 461, 462
Ammon, mur., . . 49, 51,52,53, 54, 60,
61, 205, 428
PAGE
Anacardium.......8,9,50, 51, 52, 53,
60, 61, 77, 90, 96, 201, 202, 334, 479
Anal Fissure. E. W. Berridge, M. D., 194
Angina, Follicular, Sanguinaria in . . 477
Angusturia......... 49, 51, 297, 462
Antimon, crud.,...........448,	461
Antimonium tart., . . 48,49,50,51, 52,
53,54, 94, 96, 166, 184, 224,445,447,450
Antipathies, . . ........ .' . 491
Aphorisms of Hippocrates. Bcenning-
hausen’s. Trans, by A. McNiel, M.
D.,............. 99,116,161,188, 242
Apis ; Clinical Symptoms. J, E. Wi-
nans, M. D.,................. 23
Experience with; a Clinical Note.
Wm. A. Hawley, M. D„........133
Apis. 51, 52, 53, 96,193, 240, 258, 333,335,
374, 389, 406, 445, 448,453
Apocynum, ............ 49, 335
Apoplexy,..................  242
Aqua Fortis, ............. 354
Archives of Gynaecology for July,
Notice of,.............. 348
Argent, ......... 49, 50,53, 407, 444
Argentum nitricum. A Lecture. J. T.
Kent, M.D............... 199
Argent, nitr., . . . 52,87,126,200, 201,
202, 238, 240, 287, 377, 385, 447,449
Arnica, ...... 18,49,51, 52,53,54,
57, 58, 61. 66, 67,133; 152,154,155,156,
160,192, 208, 238, 239, 243, 278,279,330,
379, 346, 445, 448, 449, 451,453,456,459,
461, 462
Arsenicum Album. A Lecture. Prof.
A. McNeil, M. D.,...........330
Arsenicum, . . . . . 1,3, 4,11,15,49,
52, 53, 57. 75, 76, 78, 80, 81, 96,131, 135,
152,156.157,159,160, 200, 202, 204, 206,
207,208,240,243, 257, 273, 281, 328, 329,
831,332, 333,334,335, 336, 337, 344, 364,
367,374. 375, 378,379, 384, 394, 403,407,
442,446,448,450,451,454,455,461,462,
468,470,471,481
Are. hydg., ..............	51
Are. iod., ............... 384
Arsenicum metallicum, . ...... 2
Arum triphyllum........ 15,48,
51,193, 407,447.449
Arund.,............ 51,52, 60,61
Asafcet.,..............  ....	60, 61
Asar.,............... 445, 461
PAGE
Asclepias,........................15,	51
A spar.............................. 52
Asphyxia, Treatment of, in the New
Born,............................  34
Atropia, ......................... 49,51
Aurum.,.............. 10 49. 50, 52, 53,
60,61, 280, 330,447
Avena sativa.,.....................  87
Backache, Worse from Noise. C. S.
Durand, M. D.,....................389
Badiaga,...................48,51,52,429
Badiaga Case, A,..................  429
Bad Breath,.......................  351
Bad Habit. A,.......................269
Ballard. E. A., M. D. Clinical Notes
upon Parturition,.................378
Baptisia..................15, 32,113,
114,120,121,155,156,157,165,166,224,
303. 407, 445, 462
Baptisia in Hysteria. G. W. Sherbino,
M. D.,........................... 113
Barium,..........................  .	240
Baryta-carb.,............2,12.49, 52,
53, 54, 60, 61, 96,127,128,195, 208,407,
435, 458,462
Beaten at His Own Game.............. 36
Belladonna. W. S. Gee, M. D., ... 408
Belladonna,.............. 1, 8.10, 23,
49, 50, 51. 52. 53, 54, 58, 60. 61, 64, 96,
99,100,126,127,165,166,175,184, 190,
193,194,195,203, 208,211,214,240,243,
245,250. 281, 282, 328, 337,372,376,387,
406,409,410, 446,448,450,451,452,456,
458,459,461,462,467
Bender, Dr. Prosper,............ 36
Benzoic ac., ................... 337,469
Berberis,........................445,448
Berridge, E. W., M. D. A Case of Anal
Fissure, .........................194
Capsicum Cough...............390
Clinical Notes..................  65
Cyclopaedia of Drug Pathogenesy,
The Revised Version of the, . .	13
Dr. Everly’s Case in the November
Number, ...;................32
Dyspnoea on Falling Asleep, ...	48
Dr. Kent on Sycosis. ............ 23
Keynotes, and the Totality of the
Symptoms,..................... 44
Lachesis Nine-Millionth, .... 195
Lac Felinum......................123
Paralysis—Manganum...............115
Soft Corns Cured by Wiesbaden,. 477
Berberis, ........................... 194
Biegler, J. A., M. D. Clinical Cases,. 447
Gamboge Case,....................  .	428
Hay Fever,. .....................317
Bismuth,....................8,50, 52,448
Book Notices and Reviews,. . . 34,69,
104,140,180. 220, 347, 391,436
Books and Pamphlets Received, . 105,
348,438
Borax, . . 20,49, 50. 60, 61,154,206, 376, 461
Bovista, . . . 2,49, 51,52,60,61,369, 457, 485
Boxing the Ears, ......... 351
Brief, but Attractive,............  314
Bromium,. . 15,49, 50,52,114,166, 224,
333,458, 484
Brown, Titus L., M. D. In Memoriam, 390
Bruns. Dr. Frederick; Removal of,. . 220
Bryonia,. . 48, 49, 50, 52, 53. 54. 57, 60,
61,112.120,126, 153,184,185, 208, 214,
236, 238, 239, 240,282,303, 339, 387, 389,
404,407, 433,444, 445, 446, 447, 448, 450,
455,457,458. 559, 461
Bufo................. ... .'.50,51,462
Butler, Clarence Willard, M. D.
A Case of Dysentery,............246
A Happy Coincidence,............ 58
Cactus grand.,. .18,20,53,239,340,445,
447,	462
Cad. sulph...................... 48
Cainca,...........................51,	53
Caiuput,........................ 32
Calad.,...................53,54,445,461
Calc, ac.,...................... 54
Calc, carb ,. .47, 49, 50, 53, 54. 60, 61,
96 126.130, 131, 132,170,171, 174, 194,
204, 206, 207, 208,209, 211, 237, 240, 282,
329, 330.334. 344, 367, 871, 376,378. 4.33,
434.444, 446, 447, 448, 452, 458, 459, 472, 485
Calc, flu.,........................ 52
Calc, jod.,.........................354
Calcarea phos.,.... 19,' 20,51, 60, 61,472
Calendula,...................... 60,	279
Campbell, Dr. Alice B. Address at
Opening of Hering Mem. Hospital,. 414
Camphor,... 15, 50,76, 78, 80,81, 448, 485
Cane, flv.........................50,52
Canfield, Dr. Corresta T.,.........269
Cannabis indica, . . .13.175,201, 234, 354
Cantharides for Hydrophobia, .... 352
Cantharis,................... 11.407, 444
Capsicum,............5,	52, 53, 54,157, 407
Capsicum Cough. E. W. Berridge,
M D.,.............................390
Three contributions to the study of, 4
A Brief Study of. C. Carleton
Smith, M. D.....'.............. 4
The Mind and Skin of. Wm. Jef-
ferson Guernsey, M. D.,........ 7
A Few Comparisons of. John V.
Allen, M. D................... 10
Carbo an , ... 48, 49, 52, 53, 60, 61, 96, 281
Carbo veg., . . .1,2.5,6,48.49,52,53,
54,60, 61, 96,135,153,154,156,159, 204,
206, 237, 238,239, 240, 318, 335,336, 344,
375,403, 454, 474,475, 485
Carbolic acid,........... 13,179, 250
Carbn. s.,........................  54
Card, m., ......................... 53
Carlsbad,.......................... 48
Cascarilla, ....................... 68
Case for Consultation ; S. L.,. . . . .	84
Castor Oil,.....................216,	250
Catalogue and Price Current of Homoe-
opathic Goods. Otis Clapp & Sons,. 180 ’
Catarrhal Pneumonia. G. W. Sherbi-
n6, M. D.,......................  165
Cauloph.,...........................453
Caustfcum, . . 8. 9, 12.18, 47. 49. 50, 52,
53, 54, 60. 61, 67, 96,193,194, 238, 260,
280, 282,346, 369,391, 405,410, 445,454. 458
Cedron.,........................  51,	68
Central New York Homoeopathic Medi-
cal Society, Report of.. . . .91, 370, 465
Chamomilla, . 6,10.49,52, 53. 54, 58.60,
61, 88 154. 238. 282, 323. 377,448,449, 462
Characteristic. Notes upon a, . . . . 224
Notes on. C. Carleton Smith, M.
D., Edmund J. Lee, M. D.,. . . 443
Some......................    .	340
Chase, Dr.C.E,.....................269
Chelidonium, ...... 10, 49, 52, 54, 224
Chestnut............................... 33
Chimaphila, .........................60
China, or Cinchona. A Lecture by
Professor J. T. Kent, M. D.,........152
China, ... 49. 50. 51, 52. 54, 67, 78, 96,
122,126,153,154.155, 156, 157,158,159,
160, 204, 236,281, 343, 344, 384, 386, 403, 404
Chin.s,.......................49,50,473
PAGE
Chloral Eruption,..........270
Chloral Hydrate,.... 137,174,377, 440
Chloroform, ............ 31, 137
Cholera, The Cause of,..... 21
Chrom. ac,..............	61
Chronic Diseases. J. G. Gundlach, M.
D., ................ 474
Cicuta,............... 50, 867, 462
Cimex,.....................474
Cimicifuga-Corregendum. Wm. Jeffer-
son Guernsey, M. D.......... 163
Cimicifuga,......... 165,	271,272
Cina,. . 49, 50, 52, 68, 207,279, 281, 282, 444
Cinchona,..................157
Cinnab., ..............	61
Cistus can.,.........60,61,407, 410
Citr.,.................... 462
Clark, Geo.H., M.D., Correspondence, 139
Dulcamara in the Ovario—Uterine
Sphere,....................280
Tellurium, ............	60
Class-Room Talks. Prof. J. T. Kent,
MB................. 458
Clematis, ...............	3
Clinical Case. J. A. Biegler, M. D., . 447
A. B. Eadie; M. B........ 121, 248
Wm. Jefferson Guernsey, M. D., .	62
Prof. J. T. Kent, M. B.». . . . 124, 301
G. W. Sherbino, M. D...... 302, 387
Clinical Notes. E. W. Berridge, M. D., 65
Clinical Note, A. Geo. H. Higgins, M.
t)	....... . . . 390
Clinical Verifications. G.W. Sherbino,
M. D................. 339
COca, .............. 51,52, 53
Cocculus,........ 239, 240, 242, 427
Coccus C., 49,50,51,52,53, 54.185, 281
329, 344,445,446
Cocoa and Chocolate. Pub. by Walter
Baker & Co. Review of....... 105
Ooti	.	.	. . . 49.53 54
Coffea, ’ 52, 53, 54* 88,' 369’, 427, 449. 458,461
Coincidence, A Happy. Clarence
Willard Butler, M. D., .......	58
Colchicum, . .53,60,322,331,344,377,
403,404, 462
Colds. To Prevent, .......... 221
Collinsonia, ...........	447
Colocynth,. . 11,17, 50, 54, 67,128,129,
157, 281, 364
Colocynth Symptom, An Unusual. R.
Gibson Miller, M. B.,	....128
Comoc, ...... . ....... 50, 53
Conium, . . . 8, 50, 52,53, 60.115,189,
194, 281 282, 337, 367, 461, 462
Constipation, Remarkable Case of. J.
B. Elliott, M. D., .......... 429
Consultation, A Case for. S. L., . . .	84
Consumption and its Treatment. Dr.
Stammers Morrison......... 348
Convalescence, Therapeutic Notes
upon. E. J. L.,........... 344
Convallaria majalis.......... 48
Copper,...... 52, 60 61,184, 240
Corns, Soft, Cured by Wiesbaden. E.
W. Berridge, M. D , ........ 476
Correction. See Errata.
Corrosive Sublimate,........ 179
Cotton, Controlling Contagious Dis-
eases with................. 70
Cotyl.,.................... 50
Cough, Time Table. J. E. Winans, M.
D., ...............	49
Country Visitors,........... 349
Creosote,.............344,445, 462
Crocus, ................ 48
PAGE
Crotalus-hor.,........... 156, 332
Croton tig , ...... 49, 52,154, 240, 247
Croup : Its Nature and Homoeopathic
Treatment. By Hurro Nauth Roy,
L.	M. 8. Review of,..........140
Cuprum, . . 49, 50,52, 80, 81, 240,344, 346
Curar,......................	. 49, 54
Cyclamen,.............54,194, 240, 346
Cyclopaedia of Drug Pathogenesy. Ed-
itorial Notes, ..............395
The Revised Version of the. E.
W. Berridge, M. D ........	13
A. George A. Taber, M. D...297
Dr. Taber and Richard Hughes,
M. D........ ....... 399
Cypripedium, ........... 87, 88
Cysts; Trans, by A. McNeil, M. D., . 130
Daphne ind,............	67
Death from Poison Ejected by a Toad, 352
DeBaun, Edwin, M. D. Offensive Feet, 434
Deck, J Feild, M. B. A Sabadilla Case, 462
Dengue; Researches into the Etiology
of. J. W. McLaughlin, M. D., . . . 348
Detwiler. Henry. M. D. InMemoriam, 212
Diet in Hypochondriasis, ...... 351
Difficult Respiration......... 392
Digitalis, . . 2, 49, 51, 54,160. 288. 335, 462
Dioscorea,. . 16, 51. 53,54,247, 303, 340, 384
Dip oma Dispute, The, ........ 222
Diseases of the Bones and Ligaments
of the Spine, A Treatise on. By S.
M.	Cate. M. D.,......... . . 349
Diseases of the Hair and Scalp, A Prac-
tical Treatise on the. By George
Thomas Jackson, M. D. Review of, 391
Diseases of Horses, with some Re-
marks on the Pleuro-Pneumonia of
Cattie. J. Hall, Sr., M. D.,. . . . . 276
Disinfectant, The Fly the Great,. . . 314
Disinfectants in Homoeopathic Prac-
tice...................... , . 315 '
Dispensary, New Homoeopathic,. . . 492
Dolichos,...................52,	53
Drosera, . , . . . 49,50. 52, 53,54,120, 160
Drugs and Medici: es of North Amer-
ica. J. U. & C. G-Lloyd. Review of,
69, 347, 489
Dulcamara,. . 3,49,54,60,236,255,275,
281, 282
P. P. Wells, M. D.......... 274
in the Ovario-Uterlne Sphere. Geo.
H. Clark, M. D., . . ...... 280
Durand, C. 8., M .D. Backache Worse
from Noise,............. 309
Dynamization, or Dematerialization.
J. B.Sunderland, M. D. Review of, 438
Dysentery, A Case: of. Clarence Wil-
lard Butler, M. D.,......... 246
and Its Treatment. Ad. Lippe,
M. D.....................363
Dysmenorrhoear—Clinical Notes. Prof.
J. T. Kent, M. D............. 19
Dyspnoea on Falling Asleep. E. W,
Berridge, M. D............... 48
Eadie, A. B., M. D. A Clinical Case,
121, 248
Ear, Novel Methods of Treating Dis-
eases of the Middle. By Seth L.
Bishop. M. D., Notice of,.....348
Editorial Notes, . . . 223, 271,315, 353, 396
Elaps.............. 60,61, 447
Elements of Modern Domestic Medi-
cine. Henry G. Hanchett, M. D., A
Review of, ............. 436
Elliott, J. B, M. D. A Remarkab’e
Case of Constipation, ....... 429
PAGE
Empiricism to Homoeopathy, From, . 270
Epidemic Remedies withan Antipsoric 351
Epsom Salt,........................468
Ergot,......................101, 272, 354
Ergotme,.......................... 250
Errata, .... 36,70, 142, 269, 350, 391, 491
Erratic..........................  339
Errors in Drug Proving. P P. Wells,
M. D.,.......................... 355
Eryng,.......................,.../.	54
Ery. a.,.......................  60,	61
E. 8. W. Correspondence,..........214
Ether...........................137, 354
Eugen, ...........................52,54
Eupatorium Perfoliatum. A Lecture
by Professor J. T. Kent, A. M., M. D., 55
Eupat. perfol., . . 12,52, 54, 56,57, 58,
152, 472
Eupator. purp., . . ..........13,52, 68
Euphorb............................ 50
Euphrasia, .... 49,50,51, 52,96, 375, 450
Euphrasia and Cepa. Hering, . . . 251
Euthanasia—Emergencies. Professor
J. T. Kent, M. D.,	..........• . 134
Everly’s, Dr., Case. Dr.Berridge, 82, 136
Exclamation Points !! H. Hitchcock,
M. D.............................368
Experiences of a Young Homoeopath.
F E. Stoaks, M. D.,............  171
Ferran’s Inoculation Statistics, Deduc-
tions from. J. E. Winans, M. D., .	66
Ferrum, . . 11,12, 49,50,52, 53,54, 96,
204, 248, 406
Ferrum acet.,......................354
Fibroid Tumor of the Ovary,........180
Flexible Collodion,................354
Fluoric?Acid, . . 52,169, 209, 240, 344, 428
Fornias, Edward, M. D. Verifications, 169
Fort Wayne Journal of the Medical
Sciences. Notice of,..........  .	348
Gamboge,..................50, 51, 54, 428
Gamboge Case. J. A. Biegler, M. D.,. 428
Gee, Professor W. S., M. D.
Belladonna,.....................408
Spongia. Notes from an Extempo-
raneous Lecture on,............ 16
Gelsemium. A Note on,...............235
20, 61, 202, 203, 318,331,354,367, 407, 468
Give Nature a Chance,...............492
Glonome,.........................1,2, 50
Glycerine...........................146
Golden Rule, A, ....................314
Gonorrhoea, Suppression of, ..... 282
Grand Opportunity, A,...............316
Graphites, . 48, 50, 52, 53,54, 58, 60, 61,
96,194, 209, 280, 281, 282, 346,374, 375, 462
Grat.,......................49, 50, 53, 54
Grindelia robusta.................  48
Grindel squarrosa,................  48
Guajac..................	... . 184 , 344
Guernsey, Wm. Jefferson, M. D.
Capsicum, The Mind and Skin of, 7
Cimicifuga-Corrigendum, .... 163
Clinical Cases,................. 62
Natrum mur. in its Antidotal
Sphere,.......................127
Natrum sul. in Asthma, ..... 129
Periostitis-Pulsatilla,.........345
Gum Arabic,........................369
Gundlach, J. G., M. D. Chronic Dis-
eases, ........................... 474
Gymnocla, . ................. 49,52, 194
Hahnemann, Portrait of.............438
Hahnemann Association of Pennsyl-
vania, ............................425
Report of Proceedings,..........479
PAGB
Hahnemannian Homoeopathy, A Spe-
cimen of,.........................139
Hall, <L. Sr., M. D. A few Diseases of
Horses, with some remarks on the
Pleuro-Pneumonia of Cattle, .... 276
Hamamelis,.....................51,	447
Hawley, Wm. A., M. D.
Address before the Onondaga
County Hom, Med. Society,. . . 400
Experience with Apis. A Clinical
Note,.........................133
Hay Fever.' J. A. Biegler, M. D., . . 317
Hellebore,............8, 50, 60,333, 453
Hemorrhage of the Bowels. J. V. Al-
len, M. D.......................485
Hemorrhage, Nasal, to Arrest, .... 352
Hepar, . . 3, 10, 18. 49, 50, 52, 53. 54, 60,
61, 68, 96. 199, 206, 208, 209, 211, 234,
235, 238, 239, 240, 407, 433, 434, 450,
453, 454, 466
Hering.
Euphrasia and Cepa,...........251
Memorial Hospital,............410
Hesse, Dr. Hamburg Cases Treated by,
with remarks. S. L.,................24
Hide-bound. E. J. L......• .... 213
Higgins, George H., M. D. A Clinical
Note.........................390,	392
Hitchcock, H., M. D.
! !...........................368
Why these Errors?.............425
Removal of, ....................36
Holloway, J. C., M. D. A Subscriber’s
Opinion,......................  492
Home Knowledge, by Rob. A. Gunn,
Review of,......................348
Homoeopaths, Words to Young. D. C.
McLaren, B. A., M. D.........110,	209
Homoeopathic League Tracts, Notice
Of,.......................36, 105, 348
Homoeopathic League, First Annual
Report,. . .....................348
Homoeopathic Materia Medica, In-
struction to the Study of the, by C.
L. Cleveland, M. D. Notice of,. . . 348
Homoeopathic Medical Education, S.
L...............................122
Homoeopathic Medical Society of Ohio,
Annual Address read before the.
Albert Claypool, M. D. Notice of,. 349
Homoeopathic Medical Society of
Penna., Transactions of,........104
Homoeopathic Therapeutics in Dentis-
try. Walter M. James. M. D., . . . 234
Homoeopathy in our Colleges, G. W.
Sherbino, M. D..............100,	196
E. J. L., . . •...............197
Homoeopathy, in its Relation to the
Diseases of Females, and Gynaecolo-
gy. Thomas Skinner, M. D. Re-
view of, .....................  105
Homoeopathy and Gynaecology. By
Thomas Skinner, M. D. Review of, 34
Homoeopathy, Negative and Positive
Effects of, on General Medicine,. .	85
Sound. .......................221
Hopeless Case Beyond a Doubt, ...	36
Hospitals of the Women’s Homoeopa-
thic Association of Pennsylvania,
The. Walter M. James, M. D.,. •
177, 249
Ad. Lippe, M. D ..............215
Howard, Clarence C., M. D., Mania a
Potu............................174
How Experiences Differ. Editorial
Notes,......................... 223
PAGE
How to Succeed as Physicians. By
James B. Bell. M. D.,..........319
Hughes, Richard, M. D. Dr. Taber and
the Cyclopaedia of Drug Pathogenesy, 399
Human Family. The..............392
Humor of it, The. Ad. Lippe, M. D., 103
Hydatic Cyst of the Liver. 8. L., . . 342
Hydrastis,.......... .... 47, 50, 54
Hydrocyanic Acid, ...... 52,115, 156
Hyoscyamus,. . 53,54, 99,100,250,332, 457
Hypericum.............. 52,68,346, 461
Hypnotism, The Medico-Legal Aspect
Of,............................22
Icterus,................	66
Ignatia. . 1,2,7.8,12,19,25,49, 50,52,
53, 54, 59, 60, 126, 194, 238, 239, 344,
346, 406, 444, 448, 450,461,462, 471
Illicium,......................427
Index to Volume Seventh, Remarks
on,...........................492
Infection from Dairy Products, ... 391
Indiana Institute of Homoeopathy, . 106
Indigo,......................52, 53
Insanity, A Manual for Students and
Practitioners. E. C. Spetzka. M. D., 438
International Hahnemannian Asso-
ciation, ......................106
Editorial Notes,..........  224
Eighth Annual Session of,... . 252
Notice of Annual Meeting of, . . 180
Notice to Members of, ...... 180
And Work. P. P. Wells, M. D., . 313
Bureau of Clinical Medicine, . . 350
Intemation Med. Congress (Notice), . 392
Intubation of Larynx, Notice of, . . 438
Iodine,. . 2, 49, 52, 85, 86, 96,199,238,
240, 340. 343, 367, 450, 451, 462, 480
Iodide of Potassium,........... 85
Iodoform, ... ........... 324
Iowa, State University of. Hom. Med.
Department,.......... 350, 392
Ipecacuanha, . . 23,47, 49, 52, 53, 54,
58,89,152,160, 276, 328, 353, 382, 446, 461
Iris-v., ................ 448
Iron, ................. 240
Isopathy, Not, . . . . . ......221
James, Walter M., M. D. Homoeopa-
thic Therapeutics in Dentistry,. . . 234
The Hospitals of the Woman’s
Homoeopathic Association of
Pennsylvania, ....... 177, 249
A Silicea Case,...............435
Jalap, . .   .................. 62
Journal of Dietetics, The. Notice of. 106
Judge Who Knows, A, . ......,. 222
July Number. The,.......... 270
Kali Bichromicum. Ad. Lippe, M. D., 183
Kali bichrom,. . . 50,51,52,54,60,61,
185, 186, 187, 204, 207, 376, 406, 444,
448, 450
Kali brom., ..................50,	68
Kali Carbon! cum. Lecture by Prof.
A. McNeil, M. D„ ......... 236
Kali carb.. . 2,49,50.51,52,53, 54, 60,
61, 96,205, 207,237, 238, 239, 240, 260,
280, 281,334,391
Kali hydriodicum, . . 52,240,247,288, 353
Kaliphos.......... .... 68, 367
Kalmia, ..........1,2,50.52, 54
Kent, Prof. J. T., M. D. Address Before
the International Hahnemannian
Association at its Eighth Annual
Meeting, .............. 225
Lectures,. . 55, 74, 106, 152, 199,
325,374, 392,403, 452
Argentum nitricum,.............199
PAGE
Kent. Prof. J. T„ M. D.
China or Cinchona,........ 152
Class-Room Talks, ........ 458
Clinical Cases,...... 124,	301
Dysmenorrhoea, .........	19
Emergencies—Euthanasia,. . . . 134
Eupatorium Perfoliatum, . . . . 55
Sulphur,.........325,374,403, 452
Veratrum Album, ...... . .	74
Kershaw, Dr. J. Martine. “At Home,” 220
Keynotes to the Materia Medica. By
Henry N. Guernsey, M. D. Review
of, ................. 140
Keynotes and the Totality of the Symp-
toms. E. W. Berridge, M. D., . . . 44
Kimball, Dr. Samuel A. Removal, . . 391
Kreosote, . ... 17, 52,53,54, 60,281, 333
Lac. acidum, ............ 109
Lae. canin um, . . 65, 67,125,126,190,
194, 254, 302, 303, 339, 406, 410
Lac-deflor,...........125,126, 194
Lac. Felinum. E W. Berridge. M. D., 123
Lachesis, . . 6,12,17,19,47, 48, 49,50,
52, 54, 60, 62, 67, 69, 76, 82, 96, 99,127,
135, 138, 156, 168, 185, 190,239,240,
260, 281, 282, 318, 327, 335, 342,343,
344, 376, 404, 406, 410, 442,446,448,
450, 457, 461,467, 478, 483
Lechesis Nine-Millionth. E. W. Ber-
ridge, M. D., ............ 175
Lachesis and Sabadilla, A Brief Study
of. C. Carleton Smith, M. D., . . . 167
Lachnanthes,.........54, 63, 407, 444
Lactic-acid, ............. 444
Lac. vaccinum defioratum, . ... 48, 50
Lact......................54,	450
Ladies’ Aid Association, ....... 492
Latyrus, ............... 367
Lauroc, ..........	50,51, 52
Laws of Blockade, On the. Thomas
Nichol, Notice of, ......... 348
Law of the Similars the only Law of
Cure in the Treatment of the Sick.
Ad. Lippe, M. D., ......... 441
Lecture to Nurses, .......... 270
Ledum, ........... 8, 52, 54, 346
Lee, Edmund J„ M. D., Dr. Swan, and
the I. H. A.............. 176
Hidebound, .......... . 213
Homoeopathy in our Colleges, . . 197
Notes on Characteristics, .	. . 443
Therapeutic Notes upon Convales-
cence, ............. 344
Lee, John K., M. D., In Memoriam. . 476
Leggett, S. L. G., M. D., Aggravation
from Noise................461
Library of the Surgeon-General’s Of-
fice, Notice of, ....... • • • 222
Lilienthal, J. E., M. D. Points. . . . 68
Lilienthal, Dr. Samuel, ....... 221
Samuel, M. D. A Case for Consul-
tation, .............	84
Cases Treated by Dr. Hesse, of
Hamburg............	24
Hydatic Cyst of Liver....... 342
Homoeopathic Medical Education, 122
Materia Medica; What is Needed, 460
Points...............	68
Lilium tig., . . . .	238, 303, 340,884,458
Lime, Carbonate of,........240
Iodide of,. ............ 354
Lippe. Ad., M. D. Address at Opening
of Hering Mem. Hospital...... 412
The American Institute of Homoe-
opathy, ............. 117
Dysentery and Its Treatment, . . 363
PAGE
Lippe, Ad., M. D.
The Hospitals of the Woman’s
Homoeopathic Association of
Pennsylvania, ......... 215
The Humor of It,...............103
Irregular Menses,..............457
Kali Bichromicum,..............183
The Law of the Similars the only
Law of Cure in the Treatment
of the Sick....................441
Paragraph 138, . . . .......... 81
PointSj ...................• .	66
Tellurium,...................... 1
Trading upon a Name,...........338
Lithsemia. By J. W. Dowling, M. D.
Notice of,......................34ft
Lithium carb....................... 52
Liver with Two Gall Bladders, ...	36
Lobelia, ................. 2,	50, 54, 451
Logic of Failure,.................•	221
Lunar caustic,.....................285
Lycopodium,. . 1, 3. 6, 12, 19, 23. 26,
28, 47,50, 51, 52, 53, 54, 60, 61, 67, 96,
120,135, 153, 156, 160, 165, 166,189,
190,194,195,200,202,207,208, 209, 224,
238, 241, 282, 317, 318, 329 . 334, 376,
384, 387, 403, 404, 405, 406,410,444,
445, 446, 447, 454, 459, 461
Magnesia carb., . . 3,18,50, 51. 52, 53,
54, 63, 301, 369, 447, 457, 458
Magnesia mur., . . .27,28,52, 54,203,
331, 461,462
Magnesia phos.,................ 28,	254
Mag. s.,	.......................53, 54
Magnet, Early Use of, in Surgery, . . 491
Manganic acid......................160
Manganum,.... 11, 52, 204, 367, 445, 450
Manganum-Paralysis. E. W. Ber-
ridge, M. D.,..................    115
ManiaaPotu. Clarence C. Howard,
M. D.,..........................174
Manna,	. ........................194
Married,	 ........................491
Marum verum......... 52, 377, 444, 450, 462
Massachusetts Homoeopathic Medical
Society, Publications of the,.... 348
Materia Medica: What is Needed. S.L., 460
Clinical; Lectures upon. Prof. E.
A. Farrington,..............  489
McCart, T. S., V. S. Toothache of
orsC3	132
McLaren, D. C., M. D. That Sepia
Case,  ......................... 69
Words to Young Homoeopaths, 110, 209
McNeil, A., M. D. Ailanthus, . . . 456
Arsenicum Alb. A Lecture, ... 330
Translation of Boenninghausen’s
Aphorisms of Hippocrates, . 99,
116,161, 188, 242
Cysts..........................130
Lecture on Kali Carbonicum, . . 236
Prophy 1 axis from Boenninghausen, 63
Puerperal Convulsions,.........372
Silicea, . ....................202
Tetanus, ......................295
Medical Advance, The,..............106
Medical Classics,..................349
Medical Current, The,..............220
Medical Education—What is it, and
when and how is it to be attained?
P. P. Wells, M. D............... 37
Medical Societies: How to Make them
Useful. Z. T. Miller, M. D., . . . . 379
Medicines vs. Doctors,.............891
Medorrhinum,.............. 23,	24, 47, 68
Mellilotus off,..................  255
PAGE
Menses, Irregular, .......... 369
Ad. Lippe, M. D.,...................457
Menyanth........................... 60
Mephites................ 54, 60, 61
Mercer. Dr. Edward W., Removal of, . 319
Mercurius,............ 2,11, 50, 52, 60,
61, 115, 131,184, 204, 206, 208, 209, 234,
280, 281, 282, 286, 290, 344, 346,364,375,
376, 377, 394, 398,404, 406,444,446,449,
453,454, 455, 483
Merc, corr.,...............11,61, 332, 442
Merc, cyan.,.....................193,	410
Mere, sol.,................ 50,54, 60, 410
Mercury: Bichloride, .... 146, 364, 365
Mezereum...............8,9, 51.52, 53,
54, 68, 96, 241
Milk Supply of Large Cities, and the
Improper Mode in which it is Con-
ducted, The, . . . ................. 35
Miller, R. Gibson, M. D. An Unusual
Coloeynth Symptom.................  128
Miller. Z. T.. M. D. Medical Societies:
How to Make Them Useful, .... 379
Minnesota Medical Monthly, The, . . . 220
Mongrel Medical Collegeof the Future, 199
Morphia............... . 52,112,115, z
135,137,154,155, 250, 274, 303, 355, 364,
396,440, 441
Moschus..........49,	51, 52, 54, 61, 446, 448
Murex,.............................  19
Muriatic Acid,..........11,	51, 53, 54,
346, 461, 462
Mygale,.............................455
Myrt. c.,...........................54
Nash, E. B., M. D. A Proposal for a
Work on the Therapeutics of the
Throat,.............................189
Therapeutics of the Throat, . . . 406
Natrumars., . . .......... 49,50,52,447
Natrum carb., . . .......... 3, 20, 49,
50, 51, 52, 60. 346, 461
Natrum mur., in its Antidotal Sphere.
Wm. Jefferson Guernsey, M. D., . . 127
Natrum mur.............i, 2,16, 49, 50,
52, 53, 54, 56,60, 87, 128, 152. 194, 199,
200, 206, 321, 333, 405, 444, 446, 448
Natr. phos.,.......................  47
Natrum sul. in Asthma. Wm. Jeffer-
son Guernsey, M. D.,..............129
Natrum sulph.,......... 23,49.51,54,
129, 447, 462
Naja............................51.52,51
New Jersey State Homoeopathic Medi-
cal Society, ...................... 269
New York Society for Medico-Scien-
tific Investigation, Inaugural Ad-
dress before. W. Y. Cowl, M. D., . 346
Nickel-Plated Cooking-Vessels, . . . 350
Niccolum,......................51, 53, 54
Nitric acid,..........3, 49, 50, 52, 53,
54, 60, 61, 95, 96,101,194,199, 238, 240,
241, 371, 407, 410, 445, 446.447, 448, 462
Nitrum, .............49,50, 51, 54,58, 446
Nitroglycerine,....................  20
Noise, Aggravation from. S. L. G.
Leggett...........................461
Sound and,......................461
Notes and Notices,....... 34,	70,106,
143,180, 220, 269, 314. 391, 394,438
Notice, Special, ................... 70
Nux-mosch., ...... .48,52,114, 281
Nux Vomica Cases. H. E. Potter, M.D., 385
Nux vomica, . . 5. 49, 50, 51, 52, 53, 54,
67, 68, 86, 161, 186, 211, 245, 246, 277,
279, 296, 344, 377 378, 385, 386, 387,
404,407, 444, 455,456,462, 468,470
PAGE
Offensive Feet. Edwin deBaun, M.
D............................434
Oleander,..............2,52,239, 445
Oleum Animate. Julius Schmitt, M.D.,
51, 52, 54, 803
Old and New Symptoms....... 395
Old Style,....................491
Omiopatia Hahnemanniana E.Omlopa-
tiaMeticcia del Dott. Attilio Mattoli.
Follicular Amygdalitis. A. Jacobi,
M. D........................849
Only Something in It,.........221
Onondaga County Hom. Med. Society,
Address Before. Wm. A. Hawley,
M. D.,.......................400
Opiates; The Curse of,........440
Opium, ... 48, 49, 50,51, 54,115,146,
201, 212, 214, 295, 387, 838, 365,397,
432, 442,446, 447, 449,462
Opprobrium Medicorum,. .......392
Ostracism. P. P. Wells, M. D., ... 181
Over-Dosing,..................314
Oxal-ac..................18, 52, 448
Oxygen,.......................87
Oxygen in Therapeutics. By C. E.
Ehinger, M. D. Review of, ... . 436
Pacific Record of Medicine and Phar-
macy, The............ . 106
Paragraph 138. Ad. Lippe, M. D.,. .	81
Paralysis, Ascending, after Whooping
Cough. S. L., ........... 365
Paris,......................49,	53
Parturition, Clinical Notes upon. E. A.
Ballard, M. D.,.............378
Pathology and Physiology of Diabetes.
Prosper Bender, M. D.,. ...... 437
Periostitis—Pulsatilla. Wm. Jefferson
Guernsey, M. D.,.......... 345
Persistent Pain after Abdominal Sec-
tion. By James B. Hunter, M. D.,. 349
Peruvian Bark, ...............152
Petroleum,. . . . 52,53,54,60, 61,239, 280
PheR, ............ 51, 52,54, 458
Phosphoric acid, . . 1,8,11,49,52,78,
87, 88, 96, 337, 346,404,462
Phosphorus...... 10,32,49, 50, 51,
52, 54, 60, 79, 96, 120, 135, 156,159,
166, 185,194,195, 202,204,205,206,207,
210, 224, 237, 238, 240, 262, 263, 329,
331, 332, 334, 336, 344, 367,375,406,
407,433, 434,444,445, 448, 454, 455, 462, 468
Physostygma, ........... 52, 367
Phytolacca, , . .49,158, 191,193, 281, 406
Picric acid,.............. 298
Plant Analysis as an Applied Science.
Helen C. deS. Abbott, Review of,. 437
Plaster of Paris, To Remove,..269
Platina............ 32,77,96, 446
Plumbum.............. 96, 432
Pneumonia, Baptista in. G. W. Sher-
bino,.....................  120
PodophyL,............68, 237, 877, 381
Points. J. E. Lilienthal, M. D., . . . 18
S. L., . . . .	..........	68
Ad. Lippe, M. D.,......... 66
Polyporus, .... ..........	12
Polyuria,...... •.............405
Poor Hering I ........... ,221
Porter, Dr. Philip,.......... 269
Potter, H. E., M. D. Two Nux Vomica
Cases, ..... .......... 385
Prescriber, The. John H. Clarke, M.
D.. .................. 105
President’s Address at Tenth Annual
Meeting of Detroit Medical and Li-
brary Asso, C. J. Lunday, M. D., . 106
PAGE
Principio Obbietto della Materia Med-
ica. Dr. Cigliano,........  .	489
Proceedings of the Seventh Annual
Session of the International Hahne-
mannian Association, ........ 220
Prophylaxis from Bcenninghausen.
By A. McNeil, M. D.,......... 63
Pruritus, ............... 477
Psorinum, . . 32,52,54, 60, 61, 62,203,
211,238, 239, 287, 321, 323, 344,366,375,
406,444
Ptelea, ............ .... 462
Puerperal Convulsions. Trans, by A.
McNeil, M. D............. 372
Pulsatilla,. . 1, 6,8, 9,10,12, 24,25, 48,
50, 52, 53, 54, 60, 61. 76, 79, 96.117,124.
125,153,154, 156,160,184,194,199,203,
204,211, 239, 243,260. 280, 281, 282, 286,
301, 302, 327, 339,344, 346,361, 369, 377,
378, 379, 384, 389,392.403,405, 406, 433,
444, 445,447,448,452, 453,455,458, 463,
465,478, 480,483, 485
Pulse, The,.............. 352
Quinine, Evils of, • . . . . . . . . . . 221
Quinine, 55,57, 58,137,152,156,169,250,
257, 259, 335, 342, 346, 365, 396, 397,
442, 443,453, 473
Randell, A. F. Correspondence,. . . 251
Ranunc.-bulb, . . »............ 48
RaraAvis, .............. 491
Ratanhia,.....................  58
Rectum, Diseases of, Report on. J.
M. Matthews, M. D., ........ 106
Registry for Nurses, The........ 269
Remedies, Alternation of. P. P. Weils,
M. D.,....................  .	71
Repetition, A Note upon,....... 373
Frequent, not Advantageous, . . 314
Revolution in Medicine, The. By
John H. Clark, M. D., Review of,. .	69
Rheum, .............. 52, 344
Rheumatism, Sciatic, ....... -. 478
Rhododendron, ... 3,49,54,340,344, 427
Rhus-tox,. . 3, 25, 26, 27,49, 50, 51, 52,
53, 54, 57, 60, 61, 96, 111, 121, 153,160,
184, 208, 236, 238, 239, 245, 281, 282,
330,333, 334, 340,344,389,407,433, 442,
444, 457,458,459, 468, 469,478, 479
Rimedii, Individualizzati Dottor Tom-
maso Cigliano,...............488
Rochelle Balt..................244
Rochester Hahnemannian Society. . 350
Rumex,. .	. . . 18, 49, 50, 51, 52, 54,159
Ruta, .......... 2, 53, 54, 346, 446
Sabadilla Case, A. J. Feild Deck, M.D., 462
Sabadilla, . 50,54,96, 168, 346,407, 410,
464, 465
Sabina,..............199, 328,461, 462
Salicylic Acid.................255
In Foods....................351
Sambucus, . . . . 54, 67, 445,446,447,483
Sanguinaria, . .12,51,52,54,203,353,
407, 447,462, 477
In Follicular Angina, etc.,. . . . 477
Sanicula,......................255
Saponinum, ............ 47, 52
Sarsaparilla,................50,	237
Sawyer, E. W., M. D. Sulphur Case, 336
Schmitt, Julius G., M. D. The Cen-
tral New York Hom. Med. Soc.,. 91, 465
Oleum Animale, ......... 803
Stramonium in Pneumonia, , . 243
“Sycosis,”................... 94
Schreiner, Dr. Emma, ........ 438
Sciatic Rheumatism, Case of. J. A.
Biegler, M. D., ........... 478
PAGE
Secale, . . 81, 96,185,156,157,273,335,
336, 462
Seceders Rewarded,.................222
Selenium,............3, 49,60,96, 289, 344
Senega,....................49,52,54, 445
Senecio aur......................87,	88
Sepia, . . 2,3,11,18.19, 26,27, 28,48,49,
50, 51, 52, 53, 54, 60. 61, 69, 96, 158,189,
194, 237, 238, 239,260,281,282,327, 346,
369, 376, 377, 403, 406, 444, 452, 457,
460, 461, 462
Sepia Case, That. D. C. McLaren, M.
D................................ 69
Shall the Patient Eat What He Craves,1? 223
Sherbino, G. W., M. 1). Baptista in
Hysteria,........I • • •	. . . 113
Baptisia in Pneumonia,..........120
Catarrhal Pneumonia.............165
Clinical Cases...........302,339,, 387
In August Number, ....... 390
Homoeopathy in our Colleges. 100, 196
Typhoid Fever,..................214
Silicea Case. W. M. J.,............435
A Lecture. Prof. A. McNeil, M.
D.,...........................202
Silicea,. . 8, 9, 32, 49,50, 51, 52, 53, 54,
60, 61,94, 96,154,169,179,189, 202, 203,
204, 205, 206, 207, 208, 234, 235, 240, 278,
281, 324, 331, 333, 334, 335, 344,346, 367,
371, 434, 444, 445, 447,453,454, 462, 466, 472
Silver, Nitrate of,.............128,	146
Simmons, Dr. B.,................... 35
Simmons, Fourness. Correspondence, 139
Skinner, Thomas, M. D. Correspond-
ence, ............................. 31
Small, Professor A. E., Death of, . . . 64
Smith, C. Carleton, M. D.
Capsicum, A Brief Study of, . . .	4
Notes on Characteristics,.......443
A Brief Study of Lachesis and
Sabadilla, ........... 167
Spongia Tosta,..................482
Sodium............................. 28
Sound and Noise,....................461
Southern Homoeopathic Association,
The,.......................65,391, 492
Southern Journal of Homoeopathy, . . . 314
Sowing Tares, .....................350
Spigelia............54,60, 61,96, 462, 467
Spongia, Notes from Lecture on. Pro-
fessor Gee,.....................  16
Spongia Tosta. C. Carleton Smith,
M.D..............................482
Spongia,. . .17,18,50,52,53,138,449,
450, 479, 482
Squill a,..........................450
Stannum, . . 49, 50,52, 53, 54,194, 342, 448
Staphisagria, . . 16.17,49, 50,51,52,53,
54, 68. 96, 206, 238, 280, 281, 346, 371, 473
State University of Iowa, Homoeopath,
Med. Depart.,................ 350,	392
Steinrauf, Wm., M. D. A Surgical
Case,............................433
Sterility. W. H. Wathen, M. D.,. . . 106
Stictapul.,............. 50, 52, 54,67, 319
Still Waiting,.....................314
Stoaks, F. E., M. D. Experience of a
Young Homoeopath..............  .	171
Stow, T. Dwight. Proceedings of the
Central New York Hom. Med. Soc., 370
Stramonium in Pneumonia. Julius G.
Schmitt, M. D.,..................243
Stramonium, . . 2, 8,19,32, 49,50,100,
115,238,244, 245,296, 331, 337, 341,448,
450, 462
Strontium, .......... 9,52, 54, 240
PAGE
Strychnia,.........................250
Subscriber’s Opinion. J. C. Holloway,
M. D.,.........................  492
Sulphur Case. E.W. Sawyer, M.D.,. 336
Sulphur, Lecture upon. Professor J.
T. Kent, M. D....... 325,	374, 403, 452
Sulphur, . 6,8,9,12,19, 45, 47,49,50, 52,
53, 54, 57, 60, 61, 67,101,125, 153,169,
171,179,184, 202, 203, 205, 206, 207, 211,
234,237,238, 243,246, 278, 281, 282, 294,
326,327,328,329, 335, 337, 339, 344, 367,
369,375, 376,377,397,403, 406,410,433,
434,445,447,452, 453,454, 455, 456,458,
459, 468, 469
Sulphuric Acid, . 49, 50, 52, 54, 235,256, 448
Suppositories of Ice,..............222
Surgical Bureau of the International
Hahnemannian Association, . . . 173
Surgical Case, A. William Steinrauf,
M.D.,.........................433
Swan, S., M. D. Provings of Vacinni-
num,.............................  168
Swan, Dr., and the I. H. A.,......176
Syncope in Hot Baths, Danger of,.	.	.	142
Sycosis. Julius Schmitt, M. D.,	...	94
Dr. Kent on. E. W. Berridge,
M.D........................ 33
Symphytum,......................256, 447
Symptoms Undeveloped,...........351
Syphilinum,.....................46, 123
Tabac.,......................49, 53, 445
Taber. Geo. A., M. D. A Cyclopaedia
of Drug Pathogqnesy,.............297
Dr., and The Cyclopaedia of Drug
Pathogenesy. Richard Hughes,
M.D.,...........................399
Tarentula, . . 49, 51,53, 54, 60, 61,135,
339,340,442, 447
Tartarus emeticus,..............  .	243
Taught by an Allopath. (Note,) . . . 350
Taxus, ...........................  67
Technics, Notice of, ..............220
Tellurium. Dr. George H. Clark,...	60
Ad. Lippe, M.D.................. 1
Tellurium,........ 2,3,49, 60, 61,190, 444
Tetanus. Trans. Professor A. McNiel,
M.D.,............................295
Texas Homoeopathic Med. Asso., The, 142
Therapeutic Notes upon Convales-
cence. E. J. L.,.................344
Therapeutics of the Throat, A Proposal
for a Work on the. E. B. Nash, M.
D.,........................  189,	406
Theridion,..................54,461, 462
Thuja.............3,10. 48, 49, 51, 53,
61, 87, 94, 95, 96,126,188,189, 200,201,
202, 209, 259, 279, 281,285,290,355,371,
453, 462
Tobacco........................ • • 212
Tongue, Uses of,  .................891
Toothache of Horses. T. S. McCart,
V. S., ..........................132
Trading upon a Name. Ad. Lippe,
M. D.,...........................338
Trombidium,......................11,	240
Truth Well Stated, The,............270
Tuberculosis, Another Cure for, . . . 221
Typhoid Fever. J. V. Allen, M. D.,. 487
G. W. Sherbino, M. D.,.............214
Urinalysis, Practical, with Clinical
Hints. By J. B. S. King, M.D., . . 104
Vaccininum, .......................453
Vaccininum, Provings of. S. Swan,
M.D.,.............................168	1
Veratrum Album. Prof. J. T. Kent,
M.D.............................. 74
PACT
Veratrum alb., . . . .49,53,54,68,75,
76, 77, 78, 79, 80, 81, 88, 159. 164,
203, 239, 243, 333,446, 447, 448, 453, 485
Verbascum,..................11,53,54
Verifications. Edward Fornias, M.D., 169
Flora A. WaddeU, M. D...........426
Views upon Homceopathic Practice,
Dr. Alien’s,..................896
Visiting Lists, Reviews of,.....490
Waddell, Dr. Flora A. Verifications, 426
Wanted, ............... 269
Wants the Earth,................349
Watts, F. E., M. D...........	70
Wells, P. P., M. D. Alternation of
Remedies,......................71
The American Institute and its
Laughter,...................107
Dulcamara,...................274
Errors in Drug Proving,......855
I. H. A. and Work,...........313
Medical Education—What is it,
and Where and How is it to be
Obtained?...................37
Ostracism,...................181
What is Taught?..............143
Wesselhoeft, Dr. Wm. P. Address at
Opening of Hering Memorial Hos-
pital, .........................418
PAGE
What is Taught. P. P. Wells, M. D.,. 143
Who is the Quack?. . . . ;....270
Whooping Cough, Ascending Paralysis
After. 8. L.,................365
Why these Errors? H. Hitchcock,
M. D.,.......................425
Why we Fail. Editorial Notes, . . . 353
Wiesbaden. Notes upon New Reme-
dies.........................240
Wiesbaden, Soft Coms Cured by. E.
W. Berridge, M. D.......... 477
Winans, J. E., M. D. Apis, Clinical
Symptoms,.................... 23
Cough Time-Table,...........49
Deductions from Ferrane's Inocu-
lation Statistics..........66
Worms in Eggs.................492
Xanthoxylum, ........ 190,194,484
Year’s Work, The..............439
Youngman, Dr. Copartnership Notice, 142
Zems, Wm. M., M.D. In Memoriam, 476
Zine, ....... 47,49,5L 53,54, 60,
61, 68, 123, 201, 282, 333, 369,444,445,
449, 458,459,461,462
Chloride,............. 146
Sulphate...................179
Zingiber............ 49, 50,53,54
Zizia....................54,448,462
NOTICE.
All articles for publication, books for review, business communications, etc.,
must be sent to Editors, 1123 Spruce Street, Philadelphia.
Subscribers will please notice that this Journal will be sent until arrears
are paid and it is ordered discontinued.
All subscriptions are due in advance; subscribers will therefore
please forward their subscriptions at once; let each subscriber see
that his account is settled promptly.
Subscribers are respectfully requested to make all checks
and money-orders payable to THE IIOMCEOPATJIIC
PHYSICIAN, and not to either of the Editors.
				

